# EXAMINING GUARDIANSHIP RISK OF THE UNBEFRIENDED UTILIZING A SOCIOECOLOGICAL MODEL TO IMPROVE ADVOCACY AND POLICY

**DOI:** 10.1093/geroni/igae098.3247

**Published:** 2024-12-31

**Authors:** Solange Marcel

**Affiliations:** Hunter College, CUNY, New York, New York, United States

## Abstract

**Background:**

Adults without decisional capacity, having no surrogate, are known as unbefriended persons and are often referred for guardianship. Guardianship is meant to serve as a means of protection; however, a guardian’s decision-making authority is conversely a threat to autonomy. The aim of this literature review is to examine the prevalence of unbefriended persons at risk for guardianship while exploring the gaps, trends, and inconsistencies related to healthcare provider and/or institutional knowledge deficits.

**Methodology:**

A literature review was performed using electronic databases CINAHL, PubMed, Medline, and PsychInfo. Publication parameters were set between 2010 and 2024. Additionally, relevant articles with a special medicolegal focus were hand-selected without publication date restrictions.

**Results:**

Search terms resulted in 258 articles for title and abstract screening. Utilizing the socioecological model, 17 studies were synthesized. Neurocognitive disorders and psychotic disorders were intrapersonal antecedents. Social isolation increased vulnerability at the interpersonal level. Organizational factors included inadequate healthcare knowledge. Lack of guardian access was a notable environmental factor. At the policy level, health facilities lacked effective policies to promote ethical and timely decision-making. Conclusions: At each socio-ecological level, there are vastly different experiences for the unbefriended compared to decisional-incapacitated persons with identified surrogates. These differences should be the impetus for future education and serve as a springboard for advocacy initiatives. A multidisciplinary approach consisting of clinicians, healthcare ethics committees, and the guardianship system is compulsory to address the evolving needs of the unbefriended. Keywords: unbefriended, unrepresented, persons without proxies (PWP), decisional capacity, incapacitation, guardianship, and surrogates.

